# QTL and candidate gene identification of the node of the first fruiting branch (NFFB) by QTL-seq in upland cotton (*Gossypium hirsutum* L.)

**DOI:** 10.1186/s12864-021-08164-2

**Published:** 2021-12-06

**Authors:** Jingjing Zhang, Xiaoyun Jia, Xiaohao Guo, Hengling Wei, Meng Zhang, Aimin Wu, Shuaishuai Cheng, Xiaoqian Cheng, Shuxun Yu, Hantao Wang

**Affiliations:** 1 State Key Laboratory of Cotton Biology, Institute of Cotton Research of Chinese Academy of Agricultural Sciences, Anyang, 455000 Henan China; 2 Hebei Laboratory of Crop Genetics and Breeding, Institute of Cereal and Oil Crops, Hebei Academy of Agriculture and Forestry Sciences, Shijiazhuang, 050051 Hebei China

**Keywords:** *Gossypium*, QTL-seq, NFFB, Early maturity, Single nucleotide polymorphism, VIGS

## Abstract

**Background:**

The node of the first fruiting branch (NFFB) is an important precocious trait in cotton. Many studies have been conducted on the localization of quantitative trait loci (QTLs) and genes related to fiber quality and yield, but there has been little attention to traits related to early maturity, especially the NFFB, in cotton.

**Results:**

To identify the QTL associated with the NFFB in cotton, a BC_4_F_2_ population comprising 278 individual plants was constructed. The parents and two DNA bulks for high and low NFFB were whole genome sequenced, and 243.8 Gb of clean nucleotide data were generated. A total of 449,302 polymorphic SNPs and 135,353 Indels between two bulks were identified for QTL-seq. Seventeen QTLs were detected and localized on 11 chromosomes in the cotton genome, among which two QTLs (*qNFFB-Dt2–1* and *qNFFB-Dt3–3*) were located in hotspots. Two candidate genes (*GhAPL* and *GhHDA5*) related to the NFFB were identified using quantitative real-time PCR (qRT-PCR) and virus-induced gene silencing (VIGS) experiments in this study. Both genes exhibited higher expression levels in the early-maturing cotton material RIL182 during flower bud differentiation, and the silencing of *GhAPL* and *GhHDA5* delayed the flowering time and increased the NFFB compared to those of VA plants in cotton.

**Conclusions:**

Our study preliminarily found that *GhAPL* and *GhHDA5* are related to the early maturity in cotton. The findings provide a basis for the further functional verification of candidate genes related to the NFFB and contribute to the study of early maturity in cotton.

**Supplementary Information:**

The online version contains supplementary material available at 10.1186/s12864-021-08164-2.

## Background

Cotton is one of the most economically important crops, and it plays an important role in China’s agricultural economy. Upland cotton (*Gossypium hirsutum* L. AADD, 2n = 52) is one of 50 *Gossypium* species and the main natural fiber crop. It is widely planted and accounts for more than 95% of global cotton production [1]. China has a large population, and the available land for planting crops is decreasing. Improving the multiple crop index and land utilization rate is crucial for maintaining or increasing agricultural production. Early-maturing cotton has a short growth period and generally shows a dwarf and compact plant architecture [2]. Early maturity is an important target trait of cotton breeding, especially with the current adjustments to the cotton planting structure and the promotion of the mechanization of cotton production. Early maturity is a very complex agronomic trait that involves budding date, growth period, flowering time, NFFB, and the height of the NFFB [3]. These quantitative traits related to early maturity are regulated by both quantitative trait loci and the environment, which are reflected in different genetic models [4, 5]. In order to improve the land competition between grain and cotton, cotton breeding for early maturity has attracted increasing attention.

The NFFB, an early-maturity trait, refers to the number of node that generates the first fruiting branch (cotyledon node not included) [3]. This trait is associated with the flowering time and provides an estimate of the relative photoperiodism [6]. The NFFB is an easily measured and dependable morphological indicator used to evaluate cotton early maturity [7, 8]. A lower NFFB of a cotton variety indicates earlier maturation. The NFFB has direct and indirect effects on the yield percentage before frost through the bud stage [9]. Because of its high heritability and correlation with the final harvested yield, the NFFB has been used as a criterion for evaluating cotton early maturity [7].

Over the past 20 years, a large number of different quantitative traits related to fiber quality, yield, drought tolerance and disease resistance have been reported. However, inadequate attention has been focused on early maturity traits, especially the NFFB of cotton [10–15]. Previous studies have identified QTLs related to early maturity traits on most of the 26 cotton chromosomes [16–21]. More than 70 QTLs associated with the NFFB of cotton have been detected through linkage mapping or association analysis [2, 6, 22–24]. These QTLs need further confirmation and may be useful for improving early maturity via molecular marker-assisted selection (MAS). There are significant negative correlations between early maturity and both fiber quality and yield [4]. It is difficult to improve maturity, yield and fiber quality simultaneously with traditional breeding methods. Therefore, it is essential to identify QTLs and genes contributing to the NFFB, and the use of markers closely linked to QTLs for marker-assisted breeding (MAB) is key to achieve the simultaneous improvement of early maturity and other traits.

NFFB is an important agricultural trait of early maturity in cotton, and it is controlled by multiple genes, with separate minor effects. The construction of genetic linkage maps using molecular markers is a reliable method to detect QTLs related to target traits. However, the construction of linkage maps is difficult and labor intensive, especially for large population. Bulked segregant analysis (BSA) is a rapid method to detect DNA markers that are closely linked to candidate genes for a specific phenotype [25, 26]. QTL-seq has been used to identify a key QTL for grain length and weight in rice using a near isogenic F_2_ population [27]. The genetic rules for Cf*-10* in tomato were investigated by combining SNP-index and Indel-index analyses and local QTL mapping using the Kompetitive Allele-Specific PCR (KASP) genotyping approach [28]. In this study, we constructed a high-generation backcross BC_4_F_2_ population to create a highly homozygous background genotype, and we selected extreme plants based on the NFFB of the offspring to identify related QTLs. Two DNA bulks of offspring (20 individuals each) showing extremely high or extremely low NFFB phenotypic values were constructed. QTLs related to the NFFB were identified using whole genome resequencing of the two DNA bulks, and two candidate genes associated with the NFFB were predicted and verified using qRT-PCR and VIGS. These results lay a foundation for analyses of the genetic mechanisms underlying cotton earliness and the breeding of improved early-maturing cotton cultivars in the future.

## Results

### Identification of extreme phenotypic and construction of two DNA bulks

**Fig. 1:**
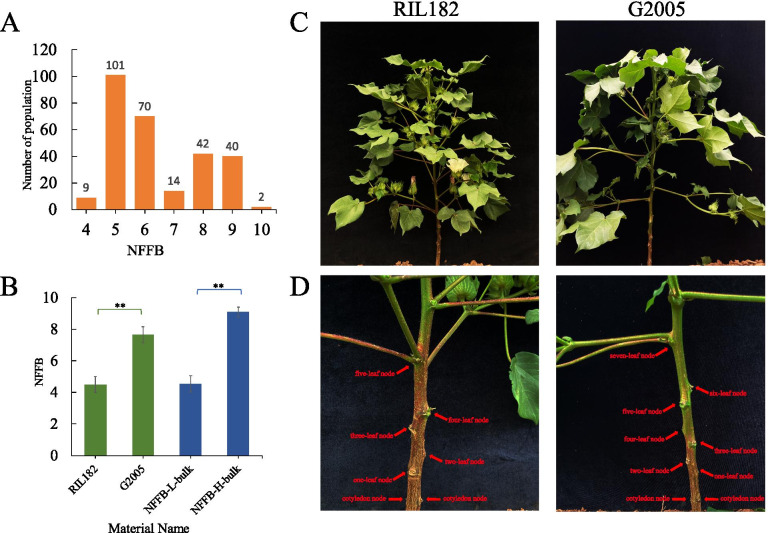
The NFFBs of the BC_4_F_2_ population, two parents, and two bulks. **a** The frequency distribution of NFFBs in the BC_4_F_2_ population; **b** Significance testing of the differences in NFFB in the two parents and two bulks. **, differences at *p* < 0.01; **c**-**d** The plant type and NFFBs of RIL182 and G2005

The average NFFB of the early-maturing cotton material RIL182 and the late-maturing cotton material G2005 were 4.5 and 7.65, respectively. Significant phenotypic differences between these two materials were observed (Fig. 1b). There were also significant differences in plant type between these two parents: RIL182 was compact, whereas G2005 was loose (Fig. 1c). The NFFB differed between the two materials (Fig. 1d). The distribution of NFFB among the BC_4_F_2_ population in the field during the budding stage is shown in Fig. 1a. Based on the results of the phenotypic investigation, two DNA bulks with average NFFBs of 4.55 and 9.1 were constructed for QTL-seq via the selection of extreme individuals from the BC_4_F_2_ population (Fig. 1b).

### Quality evaluation of Illumina sequencing data

The results of Illumina sequencing and related data analyses are shown in Table 1. High-throughput sequencing of the NFFB-H-bulk and NFFB-L-bulk generated a total of 120.77 Gb of clean data, with 61.47 Gb from the NFFB-H-bulk (Clean Q30 Bases Rate, 92.58%) and 59.30 Gb from the NFFB-L-bulk (Clean Q30 Bases Rate, 92.00%). For the donor parent RIL182, the total amount of clean data was 61.50 Gb (Clean Q30 bases rate, 92.61%). For the recurrent parent G2005, the total amount of clean data was 61.53 Gb (Clean Q30 bases rate, 92.75%) (Table 1). After trimming and filtering, over 98% of the clean reads were selected and aligned to the reference genome. The contrast ratios of all genome samples were between 98.85 and 99.48%, and the average sequencing depths for the four samples (excluding N area) ranged from 24.09 to 25.61. The the proportion of regions detected at least once in the reference genome was greater than 82.99%. The Illumina sequencing data for each sample were sufficient, and the sequencing quality was acceptable. Most of the library fragments were 350 bp (Additional file 1: Fig. S1), which was consistent with our expectations. These results suggested that the sequencing results met the criteria for mutation detection and analysis.

**Table 1:** The statistics of quality and depth for sequencing results ^(a)^

Sample	Clean Bases (bp)	Clean Reads	Clean Reads Rate (%)	Q_**30**_ (%)	Mapped Reads	Mapping Rate (%)	Mean Depth (×)	Coverage Rate (%)
NFFB-H	61,468,488,900	409,789,926	98.69	92.58	405,495,223	99.18	25.61	83.19
NFFB-L	59,301,962,100	395,346,414	98.51	92.00	393,298,443	99.48	24.09	83.20
G2005	61,529,194,800	410,194,632	98.17	92.75	405,495,223	98.85	24.57	82.99
RIL182	61,496,797,800	409,978,652	98.21	92.61	407,011,110	99.28	24.89	83.04

^(a)^ Clean bases: the reliable data after filtering; Clean reads: the reliable reads after filtering; Q_30_ (%): the percentage of the phred values greater than 30 bases in the overall base; Mapped reads: the number of reads aligned to the reference genome; Mean depth (×): the average depth of sequencing; Coverage rate (%): the proportion of regions detected at least once in the reference genome

### Detection of mutation sites

**Fig. 2:**
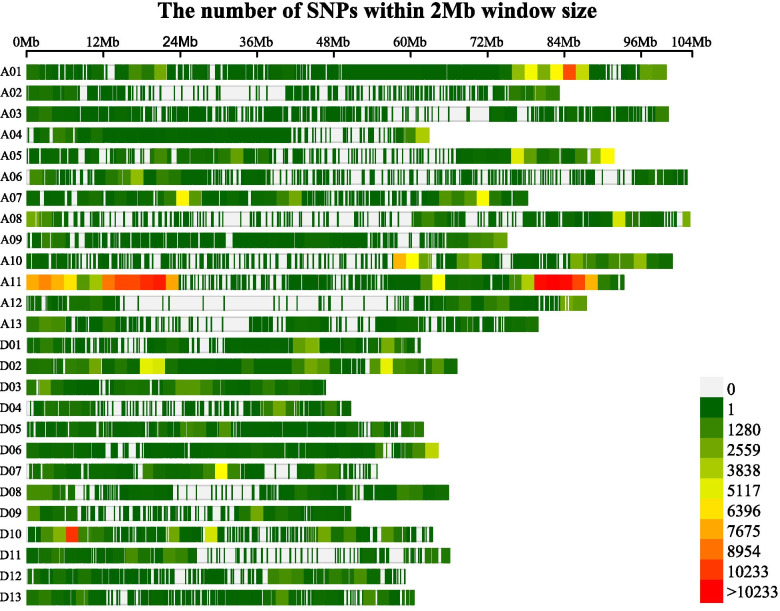
The chromosomal distribution of 449,302 polymorphic SNPs and 135,353 Indels for QTL-seq analysis in the cotton genome

When the clean reads were aligned to the TM-1 reference genome [29], two types of polymorphic markers, SNPs and Indels, were identified between RIL182 and G2005 and between NFFB-H-bulk and NFFB-L-bulk. After filtering and screening according to quality value, depth, repeatability, neighboring distance, and homozygosity of detected mutation sites, a total of 2,942,211 SNP and 391,699 Indel markers were selected, which covered all 26 chromosomes. The SNP-index of the two bulks was calculated and analyzed. A total of 449,302 polymorphic SNPs and 135,353 Indels were obtained for QTL-seq by further filtering (Additional file 2: Table S1), and the chromosomal distribution of these markers was illustrated in Fig. 2. The highest number of markers were identified on chromosome A11 (135,154 SNPs and 25,249 Indels), and the lowest numbers were detected on chromosome A03 (1689 SNPs and 3781 Indels).

### QTL-seq analysis and colocalization with previous studies

**Fig. 3:**
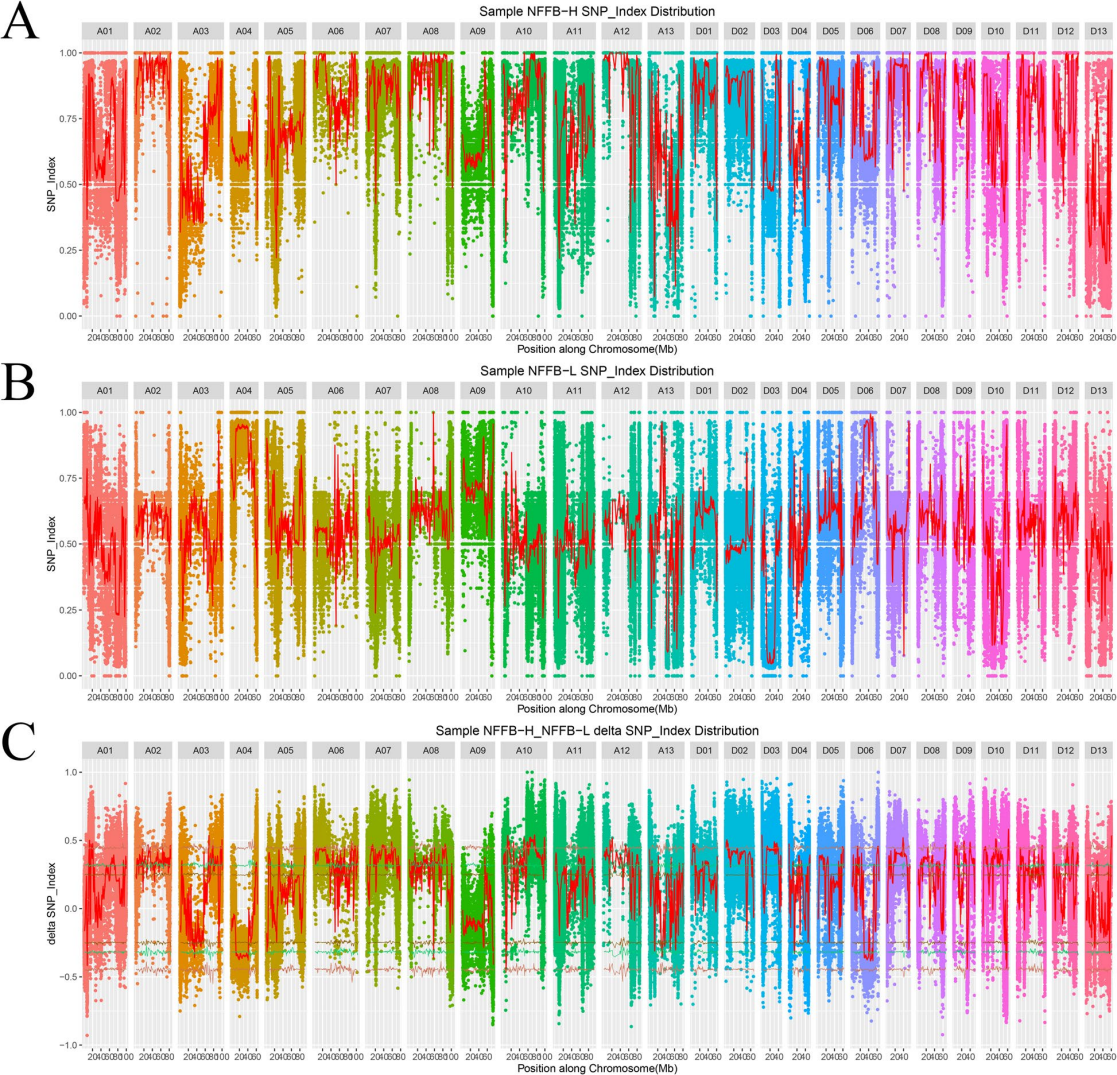
QTL-seq analysis for the identification of candidate regions on NFFB. **a** SNP-index plots of NFFB-H-bulk. **b** SNP-index plots of NFFB-L-bulk. **c** ∆SNP-index graph of all chromosomes. Statistical confidence intervals under the null hypothesis of no QTL (brown, *P* < 0.1; green, *P* < 0.05; pink, *P* < 0.01). Red plots represent the mean SNP-index and ∆SNP-index in each window

We set the window size as 2 Mb and the step size as 100 K, and the mean SNP-index and mean ∆SNP-index in each window were calculated (Fig. 3a-c). The confidence intervals of the ∆SNP-index were obtained using confidence levels of 0.1, 0.05 and 0.01 (Fig. 3c). We selected the confidence interval with a confidence level of 0.01 and the locations outside the interval were considered to be candidate regions of QTL-seq. Fifty-three regions were obtained covering 21 chromosomes, i.e., all chromosomes except A04, A05, A09, A11 and A13 (Additional file 3: Table S2). To obtain reliable QTLs, we collected most of the QTLs related to the NFFB detected in previous studies [2, 6, 22–24]. More than 70 QTLs associated with the NFFB of cotton were reported previously using linkage mapping and association analysis. The specific physical positions of these QTLs were obtained by a BLAST search against the reference genome, and were compared with the 53 QTLs detected in the present study (Additional file 3: Table S2). Seventeen QTLs overlapped with the previous results and were located on chromosomes A06, A10, A12, D01, D02, D03, D06, D09, D10, D11 and D12 (Additional file 4: Fig. S2). Only one QTL was identified on each of chromosomes A06, A10, D01, D02, D06, D10, D11, and D12. More than 2 QTLs were detected on each of chromosomes A12 and D03, and a total of 9 QTLs were identified on chromosome D03 using different localization methods and materials.

### Identification of candidate genes by expression pattern analysis

**Fig. 4:**
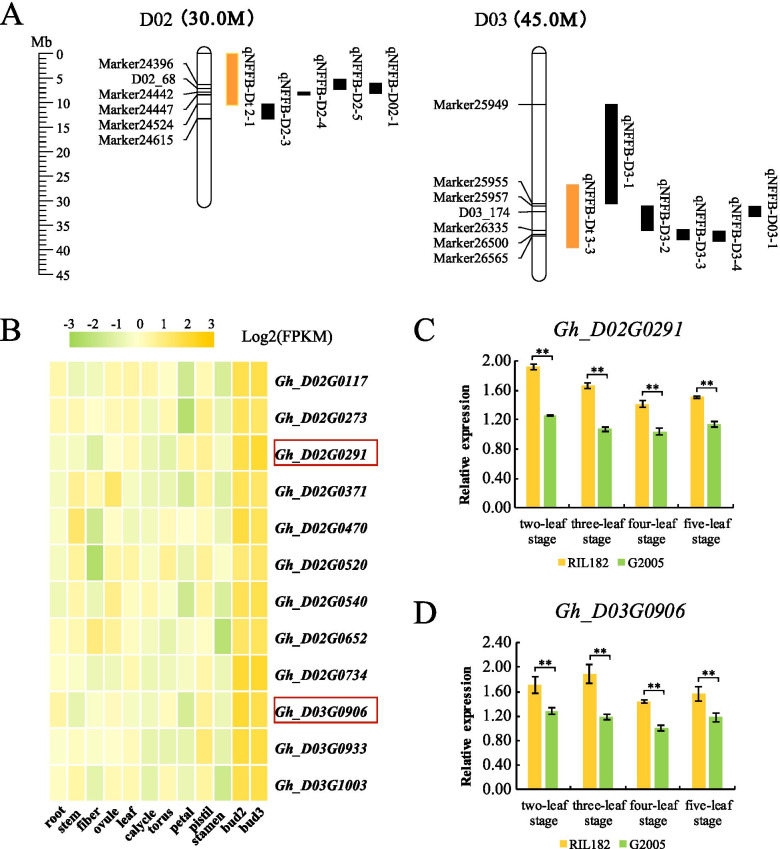
The identification of major QTLs and candidate genes related to the NFFB. **a** Two key QTLs (*qNFFB-Dt2–1* and *qNFFB-Dt3–3*) detected by colocalization. Orange regions represent QTLs detected in this study, and black regions represent QTLs detected in previous studies. **b** Expression trends of 12 genes potentially related to early maturity in 12 different cotton tissues. Scale bars on the top represent the log2-transformed FPKM values of each gene. **c**-**d** The relative expression of *Gh_D02G0291* and *Gh_D03G0906* from the two-leaf stage to five-leaf stage of RIL182 and G2005. The error bars indicate the standard deviation of three biological replicates. **, differences at *p* < 0.01

A hot spot was defined as four or more QTLs of the same trait within a 20-cM region [30]. The colocalization results showed that two QTLs (*qNFFB-Dt2–1* and *qNFFB-Dt3–3*) were located in hotspots that contained more than 4 QTLs related to the NFFB (Fig. 4a). A total of 1238 genes were located in these two candidate QTLs. To select the genes that are predominantly expressed during flower bud differentiation, the expression profiles of genes located in major QTLs in 12 different tissues were analyzed. A total of 142 genes showed higher expression at the shoot apical meristem than in other tissues; their functional annotations are shown in Additional file 5: Table S3. Twelve of these genes were selected because their homologous genes in *Arabidopsis* were related to plant reproductive development (Fig. 4b); their FPKM values in twelve tissues are shown in Additional file 6: Table S4. To examine the expression differences of the 12 selected genes between early-maturing and late-maturing cotton materials during flower bud differentiation, qRT-PCR was used to evaluate the expression differences between the two parents (RIL182 and G2005) at four different bud growth stages (two-leaf to five-leaf stages). During these four stages, the relative expression of two genes (*Gh_D02G0291* and *Gh_D03G0906*) in the early-maturing material RIL182 was significantly higher than that in the late-maturing material G2005 (Fig. 4c-d). The expression profiles of residual genes are shown in Additional file 7: Fig. S3. In addition, between RIL182 and G2005, a SNP mutation (G/A) was detected at − 989 bp upstream of the start codon of *Gh_D02G0291*, and an Indel deletion (TA/T) was detected at − 274 bp upstream of the start codon of *Gh_D03G0906* (Additional file 8: Fig. S4). These mutations may lead to differences in gene expression between the two parents. Therefore, these two genes (*Gh_D02G0291* and *Gh_D03G0906*) were considered potential candidate genes for further study.

### Gene structure and sequence analysis of *GhAPL* and *GhHDA5*

**Fig. 5:**
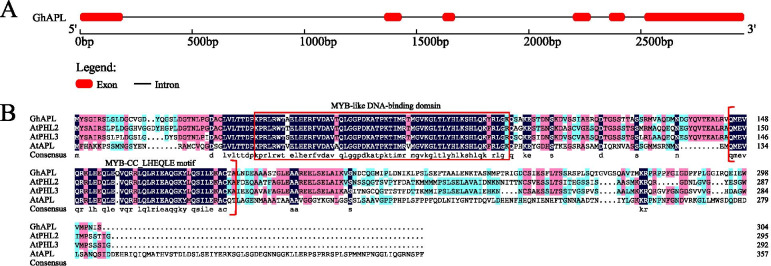
Gene structure and protein sequence analysis of *GhAPL*. **a** Structure of the *GhAPL* gene. Red boxes and black lines represent exons and introns, respectively. **b** Multiple sequence alignment of GhAPL and homologous proteins from other species. The two red boxes represent MYB-like DNA-blinding domain and MYB-CC_LHEQLE motif, respectively

**Fig. 6:**
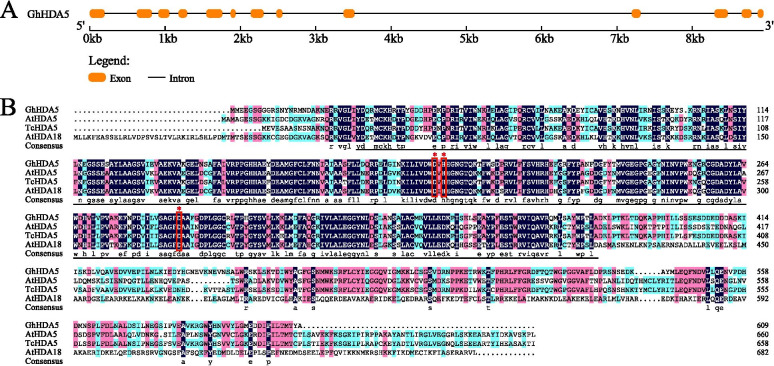
Gene structure and protein sequence analysis of *GhHDA5*. **a** Structure of the *GhHDA5* gene. Orange boxes and black lines represent exons and introns, respectively. **b** Multiple sequence alignment of GhHDA5 and homologous proteins from other species. Black lines show Hist_deacetyl domain and red boxes represent the Zn binding site

The genomic DNA and full-length cDNA sequences of *Gh_D02G0291 and Gh_D03G0906* were extracted from CottonFGD and named *GhAPL* and *GhHDA*5, respectively. The *GhAPL* gene contained 6 exons and 5 introns (Fig. 5a). The open reading frame of *GhAPL* was 915 bp in length and encoded 304 amino acid residues. The molecular mass of the GhAPL protein was 33.27 kDa, and the isoelectric point was 6.15. Comparison of the protein sequences of GhAPL with its related proteins demonstrated that GhAPL was 68% homologous to AtAPL and the GhAPL protein included a conserved MYB-like DNA-binding domain and a MYB-CC_LHEQLE motif, which indicated that *GhAPL* was a MYB-CC type transcription factor (Fig. 5b). The *GhHDA5* gene contained 13 exons and 12 introns (Fig. 6a). The open reading frame of *GhHDA5* was 1830 bp in length and encoded 609 amino acid residues. The molecular mass of the GhHDA5 protein was 68.42 kDa, and the isoelectric point was 5.32. Multiple sequence alignment revealed that GhHDA5 was 64% homologous to AtHDA5 and the GhHDA5 protein included one Hist_deacetyl domain comprised of approximately 300 amino acids with a Zn binding site that coordinated two aspartates and one histidine, indicating that *GhHDA5* belonged to the RPD3-type histone deacetylases (HDACs) (Fig. 6b).

### Silencing of *GhAPL* and *GhHDA5* in cotton

**Fig. 7:**
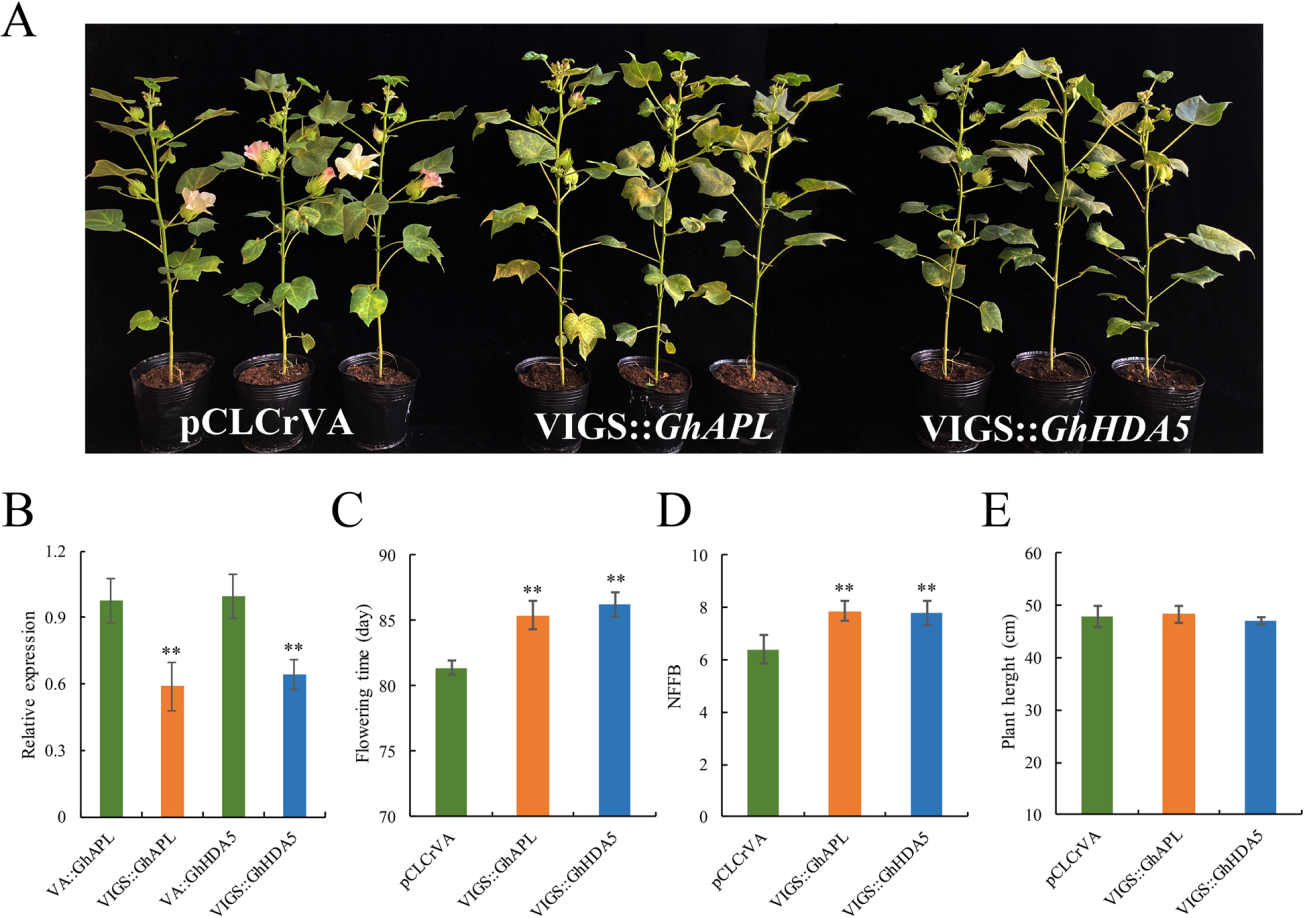
Silencing of *GhAPL* and *GhHDA5* delayed the flowering time and increased the NFFB in cotton. **a** The phenotype of pCLCrVA, VIGS::*GhAPL*, and VIGS::*GhHDA5* plants during flowering time; **b** The relative expression levels of *GhAPL* and *GhHDA5* in VA plants and silencing plants; **c**-**e** The flowering time, NFFB, and plant height of pCLCrVA, VIGS::*GhAPL*, and VIGS::*GhHDA5* plants. The error bars indicate the standard deviation of three biological replicates. **, differences at *p* < 0.01

A VIGS assay was performed to further verify the roles that *GhAPL* and *GhHDA5* play in cotton early maturity. The pCLCrVA::*GhPDS*-silenced plants had an obvious leaves whitening phenotype, which suggested that the VIGS assay was successful (Additional file 9: Fig. S5). qRT-PCR was performed to evaluate the effects of gene silencing, and the results showed that the expression levels of *GhAPL* and *GhHDA5* in positive plants were downregulated by approximately 41 and 36%, respectively, compared to the levels in control (pCLCrVA) plants (Fig. 7b). When flowering was observed in VA plants, the positive plants of VIGS::*GhAPL* and VIGS::*GhHDA5* were not flowering. The positive plants flowered later than the VA plants (Fig. 7a). We also investigated the flowering time (FT), NFFB, and plant height (PH) of VA and positive plants. The VIGS::*GhAPL* and VIGS::*GhHDA5*-silenced plants had later flowering and higher NFFB than VA plants (Fig. 7c-d). There was no significant difference in plant height (Fig. 7e). These results indicated that the silencing of *GhAPL* and *GhHDA5* delayed the flowering time and increased the NFFB in cotton.

## Discussion

Previous studies conducted with different populations and methods have detected hundreds of QTLs and several candidate genes involved in the early maturity of cotton. Among these QTLs associated with early maturity, more than 70 QTLs related to the NFFB of cotton have been identified via linkage mapping or association analysis. Traditional QTL mapping detected 23 QTLs related to the NFFB using F_2_ or F_2:3_ populations [6, 22, 23]. With the rapid development of high-throughput sequencing technology, SNPs have been applied for studies of cotton early-maturity traits. In combination with restriction site-associated DNA sequencing (RAD-seq) or genotyping by sequencing (GBS) technology, 52 QTLs related to the NFFB were identified by constructing a genetic map using SNP markers [2, 24]. Although some QTLs related to the NFFB have been identified using traditional QTL mapping, it remains difficult to perform subsequent fine mapping to elucidate the regulatory network of the NFFB when the QTLs are located in large physical intervals. Traditional QTL mapping is generally performed by genotyping a large number of individuals in segregating populations, which is labor intensive, time consuming, and costly [31]. The major advantages of QTL-seq are that the qualified SNPs filtered between the parental lines directly serve as DNA markers, and the use of SNP-index allows accurate evaluation of the frequencies of parental alleles and the genomic contributions of the two parents to the offspring [32]. The present study used QTL-seq to identify QTLs and candidate genes related to the NFFB in cotton using a BC_4_F_2_ population. Two major QTLs for the NFFB were detected by QTL-seq and colocalization, and two candidate genes related to early maturity were obtained using qRT-PCR and VIGS assays.

Through the comparison of the physical positions of QTLs related to the NFFB in previous studies, seventeen QTLs were obtained, including two key QTLs (*qNFFB-Dt2–1* and *qNFFB-Dt3–3*) that were located in hotspots related to the NFFB and were detected four and five times, respectively (Fig. 4). Among the nine QTLs, four stable QTLs (*qNFFB-D2–3*, *qNFFB-D2–4*, *qNFFB-D3–1* and *qNFFB-D3–2*) were detected in at least two years, explaining 6.33–16.33% of the phenotypic variation (PV) with LOD scores from 3.34–9.89 [24], and one major QTL (*qNFFB-D03–1*) explained 32.57% of the PV with an LOD score of 17.12 [2]. Notably, among the detected QTLs related to the NFFB, 9 QTLs were mapped using different localization methods and materials on chromosome D03 (Additional file 4: Fig. S2), which contained the most candidate regions; these results are in agreement with previous reports that chromosome D03 contains potential segments and multiple sites for early-maturity traits [19, 33]. These two key QTLs were found simultaneously by different populations and methods, which supports the reliability of the study results and lays a foundation for further fine mapping and candidate gene discovery.

Several candidate genes related to early maturity in cotton have been identified and verified in recent years. Five early maturity-related genes, *Gh_D03G0922*, *Gh_D03G0718*, *Gh_D03G0728*, *Ghir_D03G011310,* and *Ghir_A05G017290,* were identified using genome-wide association study (GWAS) and qRT-PCR, and their molecular functions were further confirmed by VIGS or genetic transformation of *Arabidopsis* [19, 33–35]. Additionally, an important regulatory gene associated with flower bud differentiation, *GhCAL*, was found by analyzing the transcript dynamics of shoot apex samples collected from two early-maturing and two late-maturing varieties, and its function was verified in transgenic plants [36]. The relative expression of all the genes detected in previous studies by qRT-PCR exhibited significant differences between early-maturing cotton varieties and late-maturing cotton cultivars during flower bud differentiation. Flower bud differentiation is a physiological and morphological symbol of the transition from vegetative growth to reproductive growth, and it has been reported that the stage of flower bud differentiation occurs earlier in early-maturing cotton than in late-maturing cotton [36, 37]. In early-maturing cotton, the flower bud differentiation generally begins at two-leaf stage or three-leaf stage [36, 38]. Therefore, qRT-PCR is a reliable method to identify candidate genes related to the early maturity of cotton during flower bud differentiation. Among the candidate genes we identified, two genes (*GhAPL* and *GhHDA5*) exhibited marked differences in expression levels between the two parents from two-leaf satge to five-leaf stage. Therefore, these genes may be associated with early-maturity traits. *GhAPL* is a homolog of *AtAPL*, which encodes an MYB-related protein in *Arabidopsis*. *AtAPL*, a key regulator of phloem development, promotes flowering through the transcriptional activation of *FLOWERING LOCUS T* (*FT*) and its transport machinery component, *FTIP1* [39]. Sequence analysis has revealed that the GhAPL protein contains a conserved SH[A/L]QKY[R/F] motif within the MYB-like domain, similar to the SHAQKYF-class of MYB-like proteins, and previous studies have shown that the SHAQKYF-class of MYB-like proteins in *Arabidopsis* play essential roles in regulating the flowering time [39, 40]. *GhHDA5*, a member of RPD3-type HDACs, is homologous to *AtHDA5*, which regulates flowering time by suppressing the expression of FLOWERING LOCUS C (FLC) and *MAF1* in *Arabidopsis* [41]. In addition, other *Arabidopsis* RPD3-type proteins have been reported to regulate the flowering time by different regulatory pathways [42–45]. The *GhHDA5*-silenced plants in our study showed obviously later flowering and higher NFFB compared to VA plants, which is consistent with the finding in a previous study that RNAi-suppressed *GhHDA5* lines showed delayed flowering [46]. These two genes were preliminarily found to be related to the early maturity of cotton in this study. However, further functional verification is necessary to clarify their specific functions.

Early maturity is a complicated physiological and biochemical process involving a large number of structural, regulatory and biochemical pathway-related genes. By identifying and cloning genes related to the NFFB, the molecular improvements in cotton maturity are performed. It is of great significance for the realization of mechanized planting, the promotion of double cropping of the cotton and wheat, and the sustained and stable development of the cotton industry.

## Conclusions

In this study, we used QTL-seq and colocalization to identify two key QTLs related to the NFFB on chromosomes D02 and D03 of the cotton genome. Then, two candidate genes (*GhAPL* and *GhHDA5*) related to early maturity were identified by qRT-PCR and VIGS assays. Both genes exhibited the higher expression levels in the early-maturing cotton material RIL182 than in the late-maturing material G2005 during the flower bud differentiation period, and the silencing of *GhAPL* and *GhHDA5* delayed the flowering time and increased the NFFB compared to VA plants in cotton. These results provide a basis for the further functional verification of candidate genes related to the NFFB and contribute to the study of early maturity in cotton.

## Methods

### Plant materials

A population comprising 137 recombinant inbred lines (RILs) was developed in a previous study from a cross between the early-maturing variety CCRI36 and the late-maturing material G2005 [24]. The NFFBs of CCRI36 and G2005 are approximately 5 and 8, respectively. Based on the phenotype and genetic composition of 137 recombinant inbred lines, RIL182 with an average NFFB of 4.5 was selected as the basic material, and the parent G2005 was used as the recurrent parent to generate BC_1_F_1_ hybrids. To increase the homozygosity of the background genotype, hybrid BC_1_F_1_ plants with lower NFFB were consecutively backcrossed to G2005 three times to create a BC_4_F_1_ population. A BC_4_F_2_ population including 278 individual plants was obtained by self-crossing for the mapping and identification of target QTLs related to the NFFB of cotton.

### Identification of extreme phenotypes and construction of two DNA bulks

The BC_4_F_2_ population, RIL182, and G2005 were planted in Anyang, Henan Province. The NFFB of cotton is the number of main stem nodes from the cotyledon node to the first fruiting branch node. We investigated the NFFB of the BC_4_F_2_ population in the field during the budding stage. According to the results of phenotypic investigation, plants with more than 8 NFFBs were considered high-NFFB plants, and plants with fewer than 6 NFFBs were considered low-NFFB plants. Two DNA bulks for QTL-seq were constructed via the selection of extreme individuals from the BC_4_F_2_ population. Twenty individuals with an average NFFB of 9.1 were sampled as the high-NFFB bulk (NFFB-H-bulk), and 20 individuals with an average NFFB of 4.55 were sampled as the low-NFFB bulk (NFFB-L-bulk).

### Extraction of genomic DNA and Illumina sequencing

Genomic DNA of the female parent RIL182, male parent G2005, and extreme individuals were extracted from the young leaf tissues using the cetyltrimethyl ammonium bromide (CTAB) method [47]. RNA enzymes were used to purify the DNA pools. The quality and concentration of DNA were examined by electrophoresis on 1% agarose gels and an ultraviolet spectrophotometer, respectively. Tested and qualified DNA samples were sent to ANOROAD company (Beijing, China). Samples with sufficient purity, concentration and volume were used to build libraries using an Illumina TruSeq DNA Sample Preparation Kit (Illumina Inc., San Diego, CA, USA). The libraries were constructed by randomly interrupting the DNA, recovering the required length of DNA fragments using electrophoresis, and adding the adapter primer. Qualified libraries were sequenced using an Illumina HiSeq 2500 sequencing platform on a computer.

### Filtering of the original data and sequencing quality control

Raw data, i.e., the original data of the Illumina high-throughput sequencing results, were stored in FASTQ files containing the name of each sequence, the base sequence and sequencing quality information. To ensure the quality of the information analysis, clean reads were generated by filtering the original data. Three steps were performed to filter the original data: (1) adapter-polluted reads (reads containing more than five bases polluted by the adapter) were removed; (2) low-quality reads (reads in which greater than 15% of the bases had a mass value lower than 19) were removed; and (3) reads with an N ratio greater than 5% were removed. After filtering the raw data, the proportion of clean reads was higher, indicating better sequencing quality. Sequencing quality was also evaluated by the GC content distribution and base mass distribution.

### Detection of mutation sites and QTL-seq analysis

Clean short reads were mapped against the TM-1 reference genome [29] using Burrows-Wheeler Aligner (BWA) software with the BWA-MEM algorithm [48]. The mutation analysis software GATK [49] was used to detect the SNPs and Indels of the population, and further filtering was performed according to some factors, such as quality value, depth and repeatability. We detected reliable mutation sites, which were annotated accordingly using ANNOVAR software [50] and an existing genome annotation file (gff/gtf). The SNP-index represents the proportion of mutant bases in the offspring, and we used QTL-seq approach to calculate the SNP-index of the two bulks [32]. To reduce the impacts of sequencing and comparison errors, the following criteria were used for SNP filtering: (1) parental genotypes were heterozygous; (2) the parents and progeny did not cover the corresponding site or the depth was less than 10; (3) the SNP-index was less than 0.3 or more than 0.7 in both bulks; (4) the SNPs were not located on main chromosomes. SNPs that met any of the criteria were removed. After filtering, the chromosomal distribution of the qualified SNPs and Indels was visualized using CMplot package based on R, and their ΔSNP-index values between two bulks were calculated by the following formula.

$$\Delta {SNP}-{index}={NFFB}-{H}\_{SNP}-{index}-{NFFB}-{L}\_{SNP}-{index}$$

A ∆SNP-index value of 1 would suggest that the locus of the bulk DNA was entirely from G2005, a ∆SNP-index value of − 1 would indicate that the locus of the bulk DNA was completely from RIL182, and a ∆SNP-index value of 0 would indicate that the two DNA bulks had the same SNP-index at this site. Therefore, an absolute value of the ∆SNP-index closer to 1 may indicate a candidate locus for the NFFB in cotton. According to the preset sliding window (the size of the window was 2 Mb, and the step size was 100 kb), the mean of the SNP-index in each window was calculated for the qualified SNPs. The confidence intervals of the ∆SNP-index were obtained under confidence levels of 0.1, 0.05 and 0.01. We selected a genetic interval with a confidence level of 0.01 and the regions outside of the interval were considered the candidate regions of QTL localization [32].

### Colocalization and candidate gene identification

To obtain reliable regions related to the NFFB, we compared our results with all of the QTLs identified in previous studies. The physical positions of markers at both ends of previously identified QTLs were found in the CottonFGD database (http://www.cottonfgd.org/) [51]. Depending on the specific physical location on the chromosome, regions that overlapped with other QTLs more than four times were considered major QTLs. To analyze the expression patterns of the genes located in major QTLs, the FPKM values of 12 tissues, i.e., root, stem, fiber, ovule, leaf, calycle, torus, petal, pistil, stamen, and buds of two-leaf and three-leaf stages, were obtained from publicly available transcriptomic data [29, 36]. The genes showing relatively higher expression during flower bud differentiation were selected, and their homologous genes in *Arabidopsis* were identified. Their functions were searched in TAIR (https://www.*arabidopsis*.org/) and STRING (https://string-db.org/) database to identify candidate genes associated with early maturity traits [52, 53].

### qRT-PCR

Total RNA was extracted from terminal buds of the RIL182 and G2005 materials at the two-leaf to five-leaf stages using a Plant RNA Purification Kit (Tiangen, China). cDNA was obtained via reverse transcription using the PrimeScript™ RT Reagent Kit (Perfect Real Time) (Takara, Japan). The composite cDNA samples were diluted 5-fold and used as cDNA templates for the qRT-PCR experiment. The qRT-PCR experiment was performed using a 7500 Real-Time PCR System (Applied Biosystems, Foster City, CA, USA) and UltraSYBR Mixture (Low ROX) (Cwbio, China) with three biological repeats and three technical replicates. The specific gene primer pairs used for PCR amplification are listed in Additional file 10: Table S5. *G. hirsutum Actin* (*GhActin*) was used as an endogenous control [54], and the 2^−ΔΔCt^ method was used to calculate the gene expression levels [55].

### Gene structure and protein sequence analysis of *GhAPL* and *GhHDA5*

The CDS, protein, and genomic DNA sequences of *GhAPL* and *GhHDA5* were extracted from the CottonFGD database [51]. The intron-exon structures of the *GhAPL* and *GhHDA5* genes were analyzed using the online software GSDS2.0 (http://gsds.cbi.pku.edu.cn/) [56]. The conserved domains within the GhAPL and GhHDA5 proteins were predicted using the online software SMART (http://smart.embl-heidelberg.de/) [57]. The Mw and pI were predicted using ExPASy (http://web.expasy.org/compute_pi/) [58]. DNAMAN 6.0 software was employed to perform multiple sequence alignments of the GhAPL and GhHDA5 proteins with their homologous proteins from other species.

### Virus-induced gene silencing

The CDS of *GhAPL* and *GhHDA5* were submitted to SGN-VIGS Tool (http://vigs.solgenomics.net/) for sequence analyses and selection of two 300-bp optimal sequences, which were amplified from the RIL182 cDNA to construct the pCLCrVA::*GhAPL* and pCLCrVA::*GhHDA5* vectors. The pCLCrVA::*GhAPL* and pCLCrVA::*GhHDA5* plasmids were transformed into LBA4404 strains. The pCLCrVA vector served as an empty control, and the pCLCrVA::*GhPDS* vector served as a positive control. The LBA4404 strains carrying pCLCrVA::*GhAPL*, pCLCrVA::*GhHDA5*, pCLCrVA (negative control), or pCLCrVA::*GhPDS* (positive control) were mixed with the strain containing pCLCrVB (helper vector) (1:1 ratio, OD600 = 1.5) and infiltrated into two fully expanded cotyledons of 10-day-old CCRI36 seedlings via syringe infiltration to generate the VA plants, pCLCrVA::*GhAPL*- and pCLCrVA::*GhHDA5*-silenced cotton, which were cultivated in a climate-controlled greenhouse with a suitable growing environment (light/dark cycle: 16 h at 28 °C/8 h at 22 °C). When the leaves of the pCLCrVA::*GhPDS* plants became white, the virus-induced silencing plants were confirmed using PCR and specific primers (Additional file 11: Table S6). The plants were transplanted into large plots and cultivated until flowering. The process of VIGS assay was the same as described previously [59].

## Supplementary Information

**Additional file 1: Figure S1**. The library fragments distribution of G2005, RIL182, NFFB-L-bulk, and NFFB-H-bulk.

**Additional file 2: Table S1**. The number of SNPs and Indels per chromosome between two bulks for QTL-seq.

**Additional file 3: Table S2**. Physical positions of 53 regions detected by QTL-seq.

**Additional file 4: Figure S2**. The distributions of 17 QTLs overlapped with the previous studies on chromosomes. The QTLs identified in this study are marked in green and red, and the QTLs located in hotspots are marked in red; The QTLs detected in previous studies are marked in gray.

**Additional file 5: Table S3**. Summary of the annotation information of 142 genes.

**Additional file 6: Table S4**. The FPKM values of 12 candidate genes in 12 cotton tissues.

**Additional file 7: Figure S3**. The relative expression of 10 genes from the two-leaf stage to five-leaf stage of RIL182 and G2005. Orange and green bar graphs show the relative expression of early-maturing cotton (RIL182) and late-maturing cotton (G2005), respectively. The error bars indicate the standard deviation of three biological replicates. *, differences at *p* < 0.05; **, differences at *p* < 0.01

**Additional file 8: Figure S4**. SNP and Indel mutations in the promoter regions of *GhAPL* and *GhHDA5* genes. The red boxes represent the specific location of mutation sites.

**Additional file 9: Figure S5**. Leaves whitening of the pCLCrVA::*GhPDS* plants.

**Additional file 10: Table S5**. Specific primers of twelve candidate genes and GhActin for qRT-PCR.

**Additional file 11: Table S6**. Specific primers used for virus-induced gene silencing assay.

## Data Availability

The raw data of DNA sequencing are available on the Sequence Read Archive https://www.ncbi.nlm.nih.gov/bioproject/PRJNA732257 under accession number PRJNA732257. The data included in this article and additional files are available.

## Abbreviations

NFFB: Node of the first fruiting branch
QTLs: Quantitative trait loci
SNP: Single nucleotide polymorphism
qRT-PCR: Quantitative real-time PCR
VIGS: Virus-induced gene silencing
MAS: Molecular marker-assisted selection
MAB: Marker-assisted breeding
BSA: Bulked segregant analysis
KASP: Kompetitive allele-specific PCR
HDACs: Histone deacetylases
FT: Flowering time
PH: Plant height
RILs: Recombinant inbred lines
CTAB: Cetyltrimethyl ammonium bromide
RAD-seq: Restriction site associated DNA sequencing
GBS: Genotyping by sequencing technology
PV: Phenotypic variation
GWAS: Genome-wide association study

## References

[1] Chen ZJ, Scheffler BE, Dennis E, Triplett BA, Zhang T, Guo W, Chen X, Stelly DM, Rabinowicz PD, Town CD (2007). Toward sequencing cotton (*Gossypium*) genomes. Plant Physiol.

[2] Li L, Zhao S, Su J, Fan S, Pang C, Wei H, Wang H, Gu L, Zhang C, Liu G (2017). High-density genetic linkage map construction by F_2_ populations and QTL analysis of early-maturity traits in upland cotton (*Gossypium hirsutum* L.). PLoS One.

[3] Fu Y, Dong C, Wang J, Wang Y, Li C (2019). Genome-wide association study reveals the genetic control underlying node of the first fruiting branch and its height in upland cotton (*Gossypium hirsutum* L.). Euphytica..

[4] Song M, Yu S, Fan S, Yuan R, Huang M (2005). Genetic analysis of main agronomic traits in short season upland cotton(*G.hirsutum* L.). Cotton Sci..

[5] Dong N, Li C, Wang Q, Ai N, Hu G, Zhang J (2010). Mixed inheritance of earliness and its related traits of short-season cotton under different ecological environments. Cotton Sci.

[6] Guo Y, Mccarty JC, Jenkins JN, Saha S (2008). QTLs for node of first fruiting branch in a cross of an upland cotton, *Gossypium hirsutum* L., cultivar with primitive accession Texas 701. Euphytica..

[7] Low A, Hesketh J, Muramoto H (1969). Some environmental effects on the varietal node number of the first fruiting branch. Cotton Grow Rev.

[8] Ray LL, Richmond TR (1966). Morphological measures of earliness of crop maturity in cotton. Crop Sci.

[9] Yu S, Huang Z (1990). Inheritance analysis on earliness components of short season cotton varieties in *G.hirsutum*. Sci Agric Sin.

[10] Fang DD, Jenkins JN, Deng DD, Mccarty JC, Li P, Wu J (2014). Quantitative trait loci analysis of fiber quality traits using a random-mated recombinant inbred population in upland cotton (*Gossypium hirsutum* L.). BMC Genomics.

[11] Tan Z, Fang X, Tang S, Zhang J, Liu D, Teng Z, Li L, Ni H, Zheng F, Liu D (2015). Genetic map and QTL controlling fiber quality traits in upland cotton (*Gossypium hirsutum* L.). Euphytica..

[12] Xia Z, Zhang X, Jia Z, Zhao H, Li C, Wang Q (2014). Major gene identification and quantitative trait locus mapping for yield related traits in upland cotton (*Gossypium hirsutum* L.). J Integr Agr.

[13] Levi A, Paterson AH, Cakmak I, Saranga Y (2011). Metabolite and mineral analyses of cotton near-isogenic lines introgressed with QTLs for productivity and drought-related traits. Physiol Plant.

[14] Ulloa M, Hutmacher RB, Roberts PA, Wright SD, Nichols RL, Michael DR (2013). Inheritance and QTL mapping of fusarium wilt race 4 resistance in cotton. Theor Appl Genet.

[15] Zhao Y, Wang H, Chen W, Li Y (2014). Genetic structure, linkage disequilibrium and association mapping of verticillium wilt resistance in elite cotton (*Gossypium hirsutum* L.) germplasm population. PLoS One.

[16] Li C, Wang X, Dong N, Zhao H, Xia Z, Wang R, Converse RL, Wang Q (2013). QTL analysis for early-maturing traits in cotton using two upland cotton (*Gossypium hirsutum* L.) crosses. Breed Sci.

[17] Li C, Song L, Zhao H, Wang Q, Fu Y (2014). Identification of quantitative trait loci with main and epistatic effects for plant architecture traits in upland cotton (*Gossypium hirsutum* L.). Plant Breed.

[18] Li C, Wang Y, Ai N, Li Y, Song J (2018). A genome-wide association study of early-maturation traits in upland cotton based on the CottonSNP80K array. J Integr Plant Biol.

[19] Su J, Pang C, Wei H, Li L, Liang B, Wang C, Song M, Wang H, Zhao S, Jia X (2016). Identification of favorable SNP alleles and candidate genes for traits related to early maturity via GWAS in upland cotton. BMC Genomics.

[20] Lacape JM, Gawrysiak G, Cao TV, Viot C, Llewellyn D, Liu S, Jacobs J, Becker D, Barroso PAV, Gibandaf M (2013). Mapping QTLs for traits related to phenology, morphology and yield components in an inter-specific *Gossypium hirsutum* × *G. barbadense* cotton RIL population. Field Crop Res.

[21] Liu R, Ai N, Zhu X, Liu F, Guo W, Zhang T (2014). Genetic analysis of plant height using two immortalized populations of “CRI12 × J8891” in *Gossypium hirsutum* L. Euphytica..

[22] Guo Y, Mccarty JC, Jenkins JN, An C, Saha S (2009). Genetic detection of node of first fruiting branch in crosses of a cultivar with two exotic accessions of upland cotton. Euphytica..

[23] Li C, Wang C, Dong N, Wang X, Zhao H, Converse R, Xia Z, Wang R, Wang Q (2012). QTL detection for node of first fruiting branch and its height in upland cotton (*Gossypium hirsutum* L.). Euphytica..

[24] Jia X, Pang C, Wei H, Wang H, Ma Q, Yang J, Cheng S, Su J, Fan S, Song M (2016). High-density linkage map construction and QTL analysis for earliness-related traits in *Gossypium hirsutum* L. BMC Genomics.

[25] Giovannoni JJ, Wing RA, Ganal MW, Tanksley SD (1991). Isolation of molecular markers from specific chromosomal intervals using DNA pools from existing mapping populations. Nucleic Acids Res.

[26] Michelmore RW, Paran I, Kesseli RV (1991). Identification of markers linked to disease-resistance genes by bulked segregant analysis: a rapid method to detect markers in specific genomic regions by using segregating populations. Proc Natl Acad Sci U S A.

[27] Qin Y, Cheng P, Cheng Y, Feng Y, Huang D, Huang T, Song X, Ying J (2018). QTL-Seq identified a major QTL for grain length and weight in rice using near isogenic F_2_ population. Rice Sci.

[28] Liu G, Zhao T, You X, Jiang J, Li J, Xu X (2019). Molecular mapping of the *Cf-10* gene by combining SNP/InDel-index and linkage analysis in tomato (*Solanum lycopersicum*). BMC Plant Biol.

[29] Zhang T, Hu Y, Jiang W, Fang L, Guan X, Chen J, Zhang J, Saski CA, Scheffler BE, Stelly DM (2015). Sequencing of allotetraploid cotton (*Gossypium hirsutum* L. acc. TM-1) provides a resource for fiber improvement. Nat Biotechnol.

[30] Said JI, Song M, Wang H, Lin Z, Zhang X, Fang DD, Zhang J (2015). A comparative meta-analysis of QTL between intraspecific *Gossypium hirsutum* and interspecific *G. hirsutum* x *G. barbadense* populations. Mol Gen Genomics.

[31] Lv Y, Guo Z, Li X, Ye H, Li X, Xiong L (2016). New insights into the genetic basis of natural chilling and cold shock tolerance in rice by genome-wide association analysis. Plant Cell Environ.

[32] Takagi H, Abe A, Yoshida K, Kosugi S, Natsume S, Mitsuoka C, Uemura A, Utsushi H, Tamiru M, Takuno S (2013). QTL-seq: rapid mapping of quantitative trait loci in rice by whole genome resequencing of DNA from two bulked populations. Plant J.

[33] Ma Z, He S, Wang X, Sun J, Zhang Y, Zhang G, Wu L, Li Z, Liu Z, Sun G (2018). Resequencing a core collection of upland cotton identifies genomic variation and loci influencing fiber quality and yield. Nat Genet.

[34] Li L, Zhang C, Huang J, Liu Q, Wei H, Wang H, Liu G, Gu L, Yu S (2021). Genomic analyses reveal the genetic basis of early maturity and identification of loci and candidate genes in upland cotton (*Gossypium hirsutum* L.). Plant Biotechnol J.

[35] Cheng X, Wang H, Wei H, Gu L, Hao P, Sun H, Wu A, Cheng S, Yu S (2021). The MADS transcription factor *GhAP1.7* coordinates the flowering regulatory pathway in upland cotton (*Gossypium hirsutum* L.). Gene..

[36] Cheng S, Chen P, Su Z, Ma L, Hao P, Zhang J, Ma Q, Liu G, Liu J, Wang H (2021). High-resolution temporal dynamic transcriptome landscape reveals a *GhCAL*-mediated flowering regulatory pathway in cotton (*Gossypium hirsutum* L.). Plant Biotechnol J.

[37] Fan L, Chen M, Dong B, Wang N, Yu Q, Wang X, Xuan L, Wang Y, Zhang S, Shen Y (2018). Transcriptomic analysis of flower bud differentiation in *Magnolia sinostellata*. Genes (Basel).

[38] Wu M, Li J, Fan S, Song M, Pang C, Wei J, Yu J, Zhang J, Yu S (2015). Gene expression profiling in shoot apical meristem of *Gossypium hirsutum*. Russ J Plant Physl+.

[39] Abe M, Kaya H, Watanabe-Taneda A, Shibuta M, Yamaguchi A, Sakamoto T, Kurata T, Ausín I, Araki T, Alonso-Blanco C (2015). FE, a phloem-specific Myb-related protein, promotes flowering through transcriptional activation of FLOWERING LOCUS T and FLOWERING LOCUS T INTERACTING PROTEIN 1. Plant J.

[40] Yan Y, Shen L, Chen Y, Bao S, Thong Z, Yu H (2014). A MYB-domain protein EFM mediates flowering responses to environmental cues in *Arabidopsis*. Dev Cell.

[41] Luo M, Tai R, Yu CW, Yang S, Chen CY, Lin WD, Schmidt W, Wu K (2015). Regulation of flowering time by the histone deacetylase *HDA5* in *Arabidopsis*. Plant J.

[42] Wu K, Zhang L, Zhou C, Yu CW, Chaikam V (2008). *HDA6* is required for jasmonate response, senescence and flowering in *Arabidopsis*. J Exp Bot.

[43] Yu C, Liu X, Luo M, Chen C, Lin X, Tian G, Lu Q, Cui Y, Wu K (2011). HISTONE DEACETYLASE6 interacts with FLOWERING LOCUS D and regulates flowering in *Arabidopsis*. Plant Physiol.

[44] Kim W, Latrasse D, Servet C, Zhou DX (2013). *Arabidopsis* histone deacetylase *HDA9* regulates flowering time through repression of *AGL19*. Biochem Biophys Res Commun.

[45] Tian L, Wang J, Fong MP, Chen M, Cao H, Gelvin SB, Chen ZJ (2003). Genetic control of developmental changes induced by disruption of *Arabidopsis* histone deacetylase 1 (*AtHD1*) expression. Genetics..

[46] Kumar V, Singh B, Singh SK, Rai KM, Singh SP, Sable A, Pant P, Saxena G, Sawant SV (2018). Role of *GhHDA5* in H3K9 deacetylation and fiber initiation in *Gossypium hirsutum*. Plant J.

[47] Porebski S, Bailey LG, Baum BR (1997). Modification of a CTAB DNA extraction protocol for plants containing high polysaccharide and polyphenol components. Plant Mol Biol Rep.

[48] Li H, Durbin R (2009). Fast and accurate short read alignment with burrows-wheeler transform. Bioinformatics..

[49] Mckenna A, Hanna ME, Sivachenko A, Cibulskis K, Kernytsky A, Garimella K, Altshuler D, Gabriel S, Daly M, Depristo MA (2010). The genome analysis toolkit: a MapReduce framework for analyzing next-generation DNA sequencing data. Genome Res.

[50] Wang K, Li M, Hakonarson H (2010). ANNOVAR: functional annotation of genetic variants from high-throughput sequencing data. Nucleic Acids Res.

[51] Zhu T, Liang C, Meng Z, Sun G, Meng Z, Guo S, Zhang R (2017). CottonFGD: an integrated functional genomics database for cotton. BMC Plant Biol.

[52] Szklarczyk D, Franceschini A, Kuhn M, Simonovic M, Roth A, Minguez P, Doerks T, Stark M, Muller J, Bork P (2011). The STRING database in 2011: functional interaction networks of proteins, globally integrated and scored. Nucleic Acids Res.

[53] Rhee SY, Beavis W, Berardini TZ, Chen G, Dixon D, Doyle A, Garcia-Hernandez M, Huala E, Lander G, Montoya M (2003). The *Arabidopsis* information resource (TAIR): a model organism database providing a centralized, curated gateway to *Arabidopsis* biology, research materials and community. Nucleic Acids Res.

[54] Gu L, Dou L, Guo Y, Wang H, Li L, Wang C, Ma L, Wei H, Yu S (2019). The WRKY transcription factor *GhWRKY27* coordinates the senescence regulatory pathway in upland cotton (*Gossypium hirsutum* L.). BMC Plant Biol.

[55] Livak KJ, Schmittgen TD (2001). Analysis of relative gene expression data using real-time quantitative PCR and the 2^-ΔΔCt^ method. Methods..

[56] Hu B, Jin J, Guo AY, Zhang H, Luo J, Gao G (2015). GSDS 2.0: an upgraded gene feature visualization server. Bioinformatics..

[57] Letunic I, Doerks T, Bork P (2015). SMART: recent updates, new developments and status in 2015. Nucleic Acids Res.

[58] Artimo P, Jonnalagedda M, Arnold K, Baratin D, Csardi G, de Castro E, Duvaud S, Flegel V, Fortier A, Gasteiger E (2012). ExPASy: SIB bioinformatics resource portal. Nucleic Acids Res.

[59] Sun H, Hao P, Gu L, Cheng S, Wang H, Wu A, Ma L, Wei H, Yu S (2020). Pectate lyase-like gene *GhPEL76* regulates organ elongation in *Arabidopsis* and fiber elongation in cotton. Plant Sci.

