# Supplementary material for: QTL and candidate gene identification of the node of the first fruiting branch (NFFB) by QTL-seq in upland cotton (Gossypium hirsutum L.)
Source: BMC Genomics. 2021 Dec 6;22:882. doi: 10.1186/s12864-021-08164-2 (PMC8650230; doi:10.1186/s12864-021-08164-2)
Supplement: Supplementary file 1 — Additional file 1: Figure S1. The library fragments distribution of G2005, RIL182, NFFB-L-bulk, and NFFB-H-bulk. [file 12864_2021_8164_MOESM1_ESM.docx]

**Additional file 1: Figure S1.** The library fragments distribution of G2005, RIL182, NFFB-L-bulk, and NFFB-H-bulk.
