# Supplementary material for: QTL and candidate gene identification of the node of the first fruiting branch (NFFB) by QTL-seq in upland cotton (Gossypium hirsutum L.)
Source: BMC Genomics. 2021 Dec 6;22:882. doi: 10.1186/s12864-021-08164-2 (PMC8650230; doi:10.1186/s12864-021-08164-2)
Supplement: Supplementary file 4 — Additional file 4: Figure S2. The distributions of 17 QTLs overlapped with the previous studies on chromosomes. The QTLs identified in this study are marked in green and red, and the QTLs located in hotspots are marked in red; The QTLs detected in previous studies are marked in gray. [file 12864_2021_8164_MOESM4_ESM.docx]

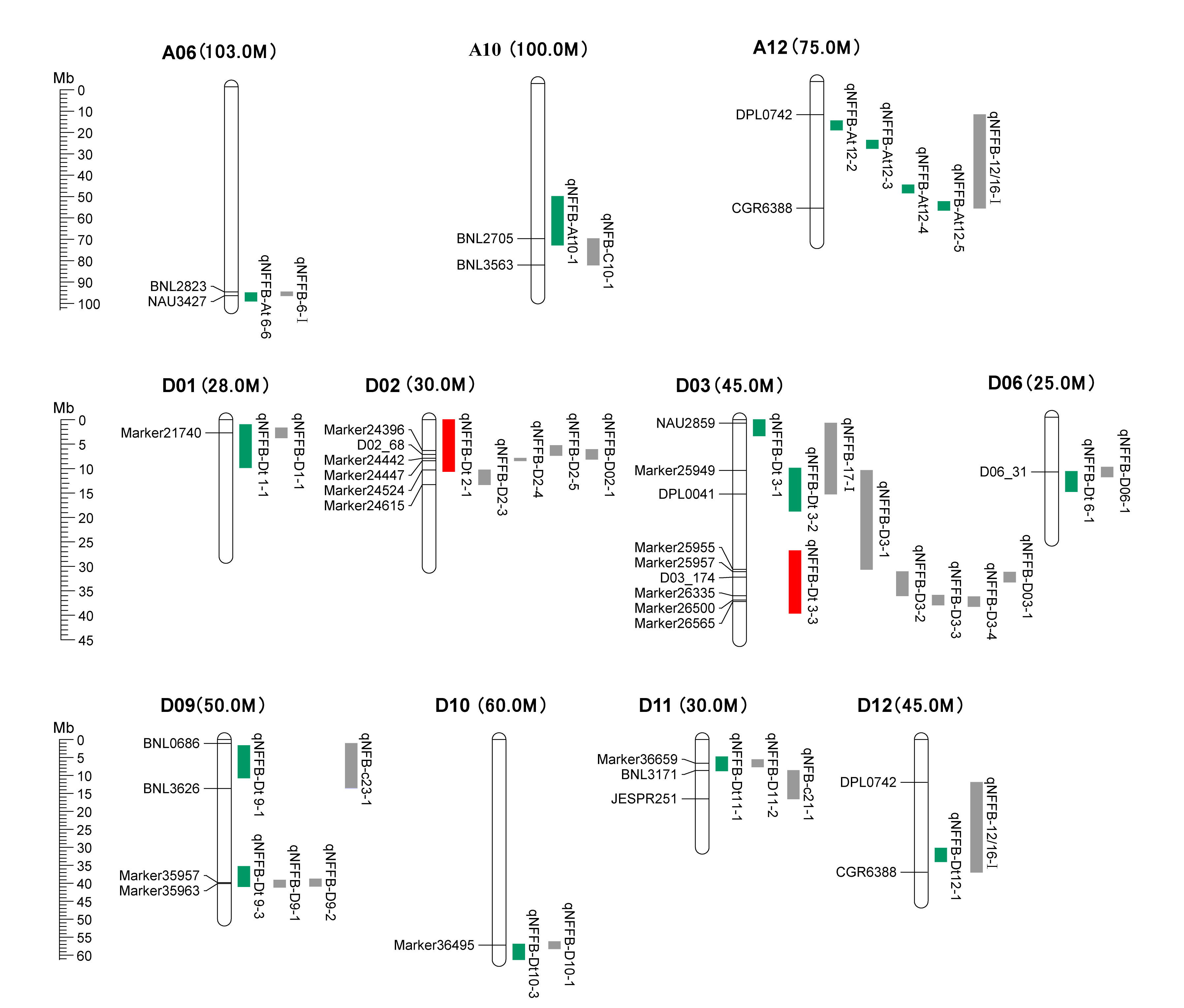
**Additional file 4: Figure S2.** The distributions of 17 QTLs overlapped with the previous studies on chromosomes. The QTLs identified in this study are marked in green and red, and the QTLs located in hotspots are marked in red; The QTLs detected in previous studies are marked in gray.
