# Supplementary material for: QTL and candidate gene identification of the node of the first fruiting branch (NFFB) by QTL-seq in upland cotton (Gossypium hirsutum L.)
Source: BMC Genomics. 2021 Dec 6;22:882. doi: 10.1186/s12864-021-08164-2 (PMC8650230; doi:10.1186/s12864-021-08164-2)
Supplement: Supplementary file 7 — Additional file 7: Figure S3. The relative expression of 10 genes from the two-leaf stage to five-leaf stage of RIL182 and G2005. Orange and green bar graphs show the relative expression of early-maturing cotton (RIL182) and late-maturing cotton (G2005), respectively. The error bars indicate the standard deviation of three biological replicates. *, differences at p < 0.05; **, differences at p < 0.01 [file 12864_2021_8164_MOESM7_ESM.docx]

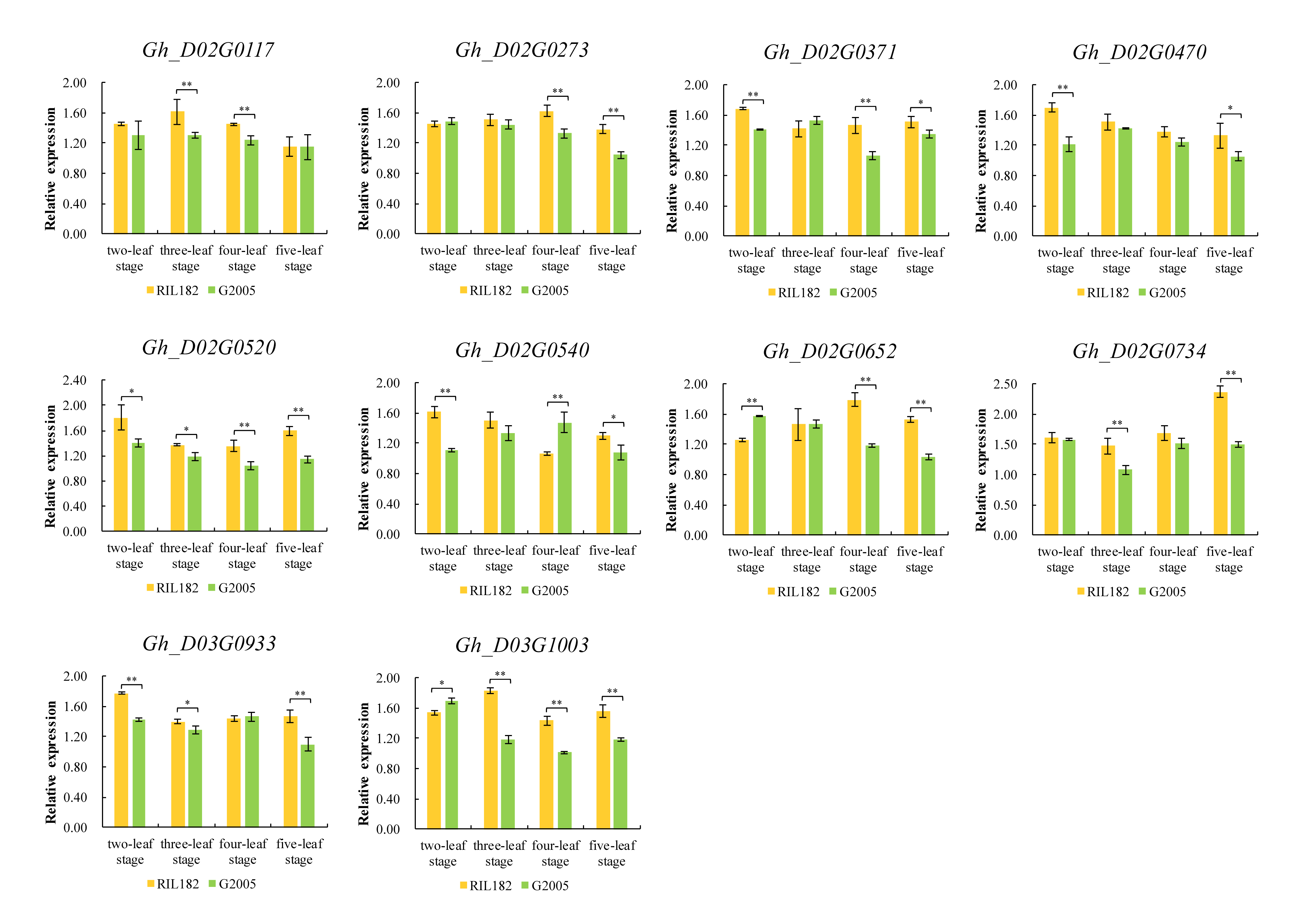
**Additional file 7: Figure S3.** The relative expression of 10 genes from the two-leaf stage to five-leaf stage of RIL182 and G2005. Orange and green bar graphs show the relative expression of early-maturing cotton (RIL182) and late-maturing cotton (G2005), respectively. The error bars indicate the standard deviation of three biological replicates. *, differences at *p* < 0.05; **, differences at *p* < 0.01
