# Supplementary material for: QTL and candidate gene identification of the node of the first fruiting branch (NFFB) by QTL-seq in upland cotton (Gossypium hirsutum L.)
Source: BMC Genomics. 2021 Dec 6;22:882. doi: 10.1186/s12864-021-08164-2 (PMC8650230; doi:10.1186/s12864-021-08164-2)
Supplement: Supplementary file 8 — Additional file 8: Figure S4. SNP and Indel mutations in the promoter regions of GhAPL and GhHDA5 genes. The red boxes represent the specific location of mutation sites. [file 12864_2021_8164_MOESM8_ESM.docx]

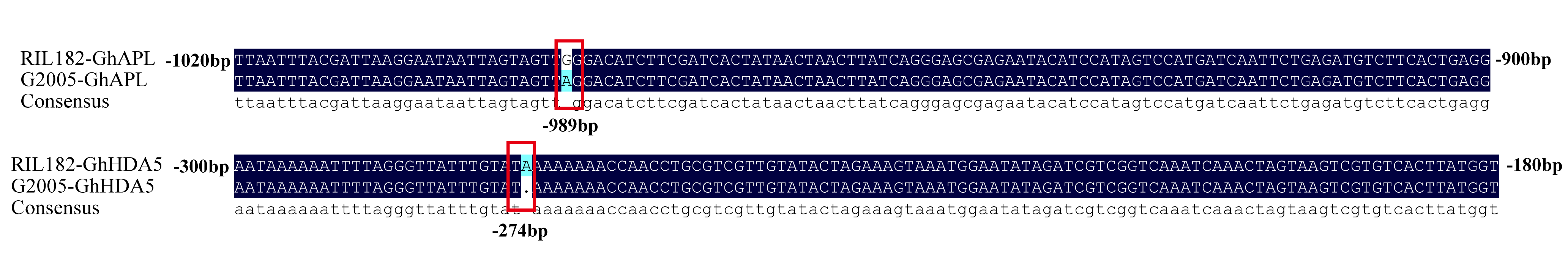
**Additional file 8: Figure S4.** SNP and Indel mutations in the promoter regions of *GhAPL* and *GhHDA5* genes. The red boxes represent the specific location of mutation sites.
