# Supplementary figures and images for: QTL and candidate gene identification of the node of the first fruiting branch (NFFB) by QTL-seq in upland cotton (Gossypium hirsutum L.)
Source: BMC Genomics. 2021 Dec 6;22:882. doi: 10.1186/s12864-021-08164-2 (PMC8650230; doi:10.1186/s12864-021-08164-2)

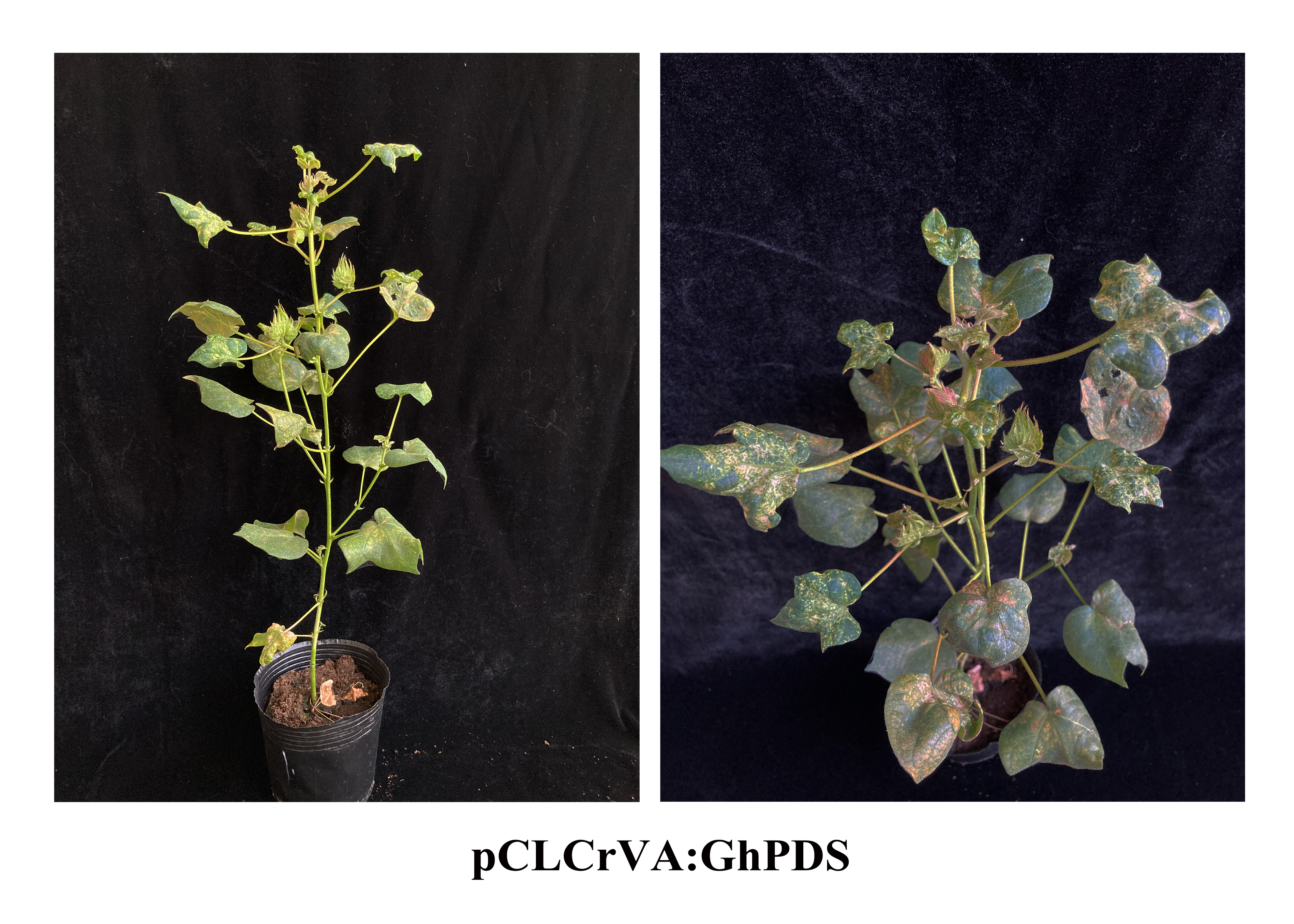
**Additional file 9: Figure S5.** Leaves whitening of the pCLCrVA::*GhPDS* plants.

Supplement: Supplementary file 9 — Additional file 9: Figure S5. Leaves whitening of the pCLCrVA::GhPDS plants. [file 12864_2021_8164_MOESM9_ESM.docx]
